# Pneumococcal surface protein A of invasive Streptococcus pneumoniae isolates from Colombian children.

**DOI:** 10.3201/eid0705.017510

**Published:** 2001

**Authors:** M C Vela Coral, N Fonseca, E Castañeda, J L Di Fabio, S K Hollingshead, D E Briles

**Affiliations:** Instituto Nacional de Salud, Bogotá, Colombia. mvela@hemagogus.ins.gov.co

## Abstract

Pneumococcal surface protein A (PspA) elicits protection in mice against fatal bacteremia and sepsis caused by genetically diverse pneumococci and protects against carriage and lung infection. We determined the PspA families of invasive isolates of Streptococcus pneumoniae recovered from Colombian children <5 years of age. That 97.5% of Colombian isolates belong to PspA families 1 and 2 supports the hypothesis that a human PspA vaccine covering a few PspA families could be broadly effective.


### Pneumococcal Surface Protein A of Invasive *Streptococcus pneumoniae* Isolates from Colombian Children

**María Claudia Vela Coral,* Nacxiry Fonseca,* Elizabeth Castañeda,* José Luis Di Fabio,† Susan K. Hollingshead,‡ and David E. Briles‡**
*Instituto Nacional de Salud, Bogotá, Colombia; †Pan American Health Organization, Washington, DC, USA; and ‡University of Alabama, Birmingham, Alabama, USA

Pneumococcal surface protein A (PspA) elicits protection in mice against fatal bacteremia and sepsis caused by genetically diverse pneumococci and protects against carriage and lung infection. We determined the PspA families of invasive isolates of *Streptococcus pneumoniae* recovered from Colombian children <5 years of age. That 97.5% of Colombian isolates belong to PspA families 1 and 2 supports the hypothesis that a human PspA vaccine covering a few PspA families could be broadly effective.

*Streptococcus pneumoniae* is a major respiratory pathogen that also causes meningitis, otitis media, and bacteremia (1). In adults, capsular polysaccharides of *S. pneumoniae* can elicit protective antibodies against pneumococcal infection (2). However, in children <2 years of age polysaccharide vaccines do not effectively elicit a protective response (3,4), and children can have repeated infections with strains of the same or different capsular serotype (5). Therefore, protein-polysaccharide conjugates and pneumococcal proteins, including pneumolysin, neuraminidase, pneumococcal surface adhesin A, and pneumococcal surface protein A (PspA), have been considered as alternative means to induce protective immunity in infants and children. The increased frequency of isolation of multidrug-resistant strains of *S. pneumoniae* accentuates the need for an effective vaccine (6).

PspA, a surface protein and virulence factor found on all isolates of *S. pneumoniae* (7), is highly immunogenic (6-8). PspAs share many cross-reactive epitopes, and immunization with a single PspA is cross-protective in mice against fatal infection with strains of the mouse virulent capsular types (6,9,10). Mucosal immunization with PspA can also elicit immunity to carriage (11,12).

Information about the basic protein structural domains of PspA came from the DNA sequences of the *pspA*/Rx1 and the *pspA*/EF5668 genes (13,14). The five domains include 1) a signal peptide, 2) an alpha-helical charged domain (amino acids 1-288), 3) a proline-rich region (amino acids 289-370), 4) a choline-binding domain consisting of 9 to 10 twenty-amino-acid repeats (amino acid 371-571), and 5) a C-terminal 17-amino-acid tail (amino acids 572-589). These amino-acid positions are based on the *pspA*/Rx1 sequence (14).

A portion of *pspA*/Rx1 (amino acids 192-260) has been identified that elicits cross-protective antibody responses (15). Many PspA molecules have been examined to aid in the development of PspA as a protein-based vaccine (16). From the alignment of PspA sequences of 24 strains, the sequence differences in a centrally located clade-defining region were used to group PspA proteins into six clades (16). Within the clade-defining region, sequences in the same clade share at least 80% amino-acid identity. The clade-defining region is roughly the same as that shown to elicit cross-protective responses (15,16). The six clades have also been grouped into three families. Sequences share at least 50% sequence identity in each family (16).

During the 1990s, the Colombian Pneumococcal Study Group investigated the capsular type distribution and antimicrobial susceptibility of invasive isolates from children <5 years of age (17). The data obtained may guide selection of polysaccharides to include in vaccines for use in Latin America. In Colombia and other Latin American countries, the prevalence of strains of capsular serotypes 1 and 5 is higher than in North America (18,19). The study demonstrated that vaccine formulations based only on North American data might not be as effective in Latin America because of the differing distributions of capsular types. Surveillance of isolates has continued to monitor any shifts in the antigenic types of pneumococci (20).

Our study was intended to expand our knowledge of vaccine coverage for a potential protein-based vaccine in Colombian isolates. We determined the frequency of family 1 and family 2 PspAs among *S. pneumoniae* isolates from Colombia that express one of the seven most common capsular types. Our results will be coordinated with those from laboratories in other Latin American countries to learn the diversity of PspA over the entire region. At present, however, only the Colombian isolates have been investigated completely.

### Materials and Methods

Forty *S. pneumoniae* invasive isolates, representing each year from 1994 through 1998, were selected from the seven most common capsular types in Colombia. All isolates were confirmed to be *S. pneumoniae* by standard procedures (alpha-hemolysis, Gram stain, optochin test, and bile solubil-

Address for correspondence: María Claudia Vela Coral,Grupo Microbiología, Instituto Nacional de Salud, Avenida Calle 26 No. 50-60, Zona 6 CAN Bogotá, Colombia; fax: 571-222 0194/3055; e-mail: mvela@hemagogus.ins.gov.co ity) (21). Antimicrobial susceptibility patterns were not considered in the selection of isolates.

Pneumococci were cultured for 6 hr in 10 mL Todd-Hewitt broth (Difco, Detroit, MI) supplemented with 1% yeast extract, 1% glucose, and 22 µg/mL glutamic acid. After centrifugation, the cells were resuspended in 0.1 mL TE (10 mM Tris HCL, 1mM EDTA, pH 8.0), and lysozyme (10 µL at 50 mg/mL) was added. The cells were incubated for 30 min at 37ºC, and 0.5 mL GES (5 M guanidine thiocyanate, 0.1 M EDTA, pH 8.0, and 0.5% sarkosyl) was added. After incubation at room temperature for 10 min, 0.25 mL (7.5 M) ammonium acetate was added, and the cells were mixed and placed on ice for 10 min. Chloroform/isoamyl alcohol (24:1) was added and mixed, the phases were separated by centrifugation, and 0.7 mL of aqueous phase was recovered. The DNA was precipitated with ethanol and resuspended in 50-100 µL of TE (22).

Polymerase chain reaction (PCR) was carried out on genomic DNA. The oligonucleotide primers for family 1 were LSM12, 5'CCGGATCCAGCGTCGCTATCTTAGGGGCTGGTT3' (23) and SKH63, 5'TTTCTGGCTCAT(C and T)AACTGCTTTC3' (at position 12 C and T in a 1:1 ratio) and for family 2 were LSM12, 5'CCGGATCCAGCGTCGCTATCTTAGGGGCTGGTT3' and SKH52, 5' TGGGGGTGGAGTTTCTTCTTCATCT3'. The following PCR conditions were used: an initial 95ºC (3 min), 30 cycles of 95ºC (1 min), 62ºC (1 min) and 72ºC (3 min), followed by 72ºC (10 min) (15) in a PTC 150 Thermocycler (MJ Research, Watertown, MA). PCR products were initially run at an annealing temperature of 62ºC. Any isolates yielding no product at 62ºC were repeated at annealing temperatures of 65ºC or 58ºC and then 55ºC to account for potential sequence divergence in the primer region. The amplified PCR products were approximately 1,000 bp for family 1 and 1,200 bp for family 2. The PCR product was run on an agarose gel at 80 volts for 1.5 hr, and the gel was stained with 0.5 µg/mL ethidium bromide. Molecular weight standard for gel electrophoresis was the 1.0-kb ladder DNA (Promega, Madison, WI). Strains BG9739 (clade 1) and AC122 (clade 3) were used as controls for family 1 and 2 tests, respectively (16). The PspA family of these strains has been confirmed by DNA sequence of their *pspA* genes (16).

A pool of immune sera for typing PspA families came from two rabbits, one immunized with rPspA/L82016 (clade 1) and the other with rPspA/Rx1 (clade 2). The antisera for typing PspA family 2 came from a pool of serum from two rabbits immunized with either PspA/V-024 (clade 3) or PspA/V-032 (clade 4). Recombinant PspA/Rx1 was added to this pool to reduce cross-reactivity with family 1 (clade 1 and 2) PspAs.

PspAs were recombinant products from *Escherichia coli* strains bearing plasmids with cloned *pspA* genes. The region of PspA in the cloned fragment includes the entire alpha-helical region of the protein and in some cases some of the proline-rich region, which is C-terminal to the alpha-helical region. The gene fragments were cloned into pET-20b vector from Novagen (Madison, WI) between the *Nco*I and the *Xho*I cloning sites. This procedure results in an additional 10 amino acids on the C-terminus of the recombinant protein, which includes a polyhistidine for purification. Recombinant proteins were produced in *E. coli* strain BL21 (DE3) from Novagen and purified by nickel-affinity chromatography, as recommended by the manufacturer. Rabbits were immunized subcutaneously with 10 µg of protein with complete Freund's adjuvant, followed by a second 10-µg injection 1 month later with incomplete Freund's adjuvant and a final 10-µg booster after another month. Rabbits were bled 2 weeks after the last booster.

Pneumococcal isolates were cultured for 6 hr in 10 mL Todd-Hewitt broth (Difco) supplemented as described. After centrifugation, cells were resuspended in 3 mL sterile phosphate-buffered saline (PBS), and 500 µL lysis buffer (0.1% sodium deoxycholate, 0.01% sodium dodecyl sulfate, and 0.15M sodium citrate in 100 µL deionized water) was added. The mixture was incubated at room temperature for 30 min.

Protein in lysates was measured by using bicinchoninic acid as described by Smith et al. (24) with some modifications. The lysate samples were diluted 1:3.5 in sterile PBS. Then, 10 µL of each sample was loaded into a microplate well (U bottom, Becton-Dickinson, Cockeysville, MD) and 200 µL of a 50:1 mixture of reagent A (bicinchoninic acid [BCA-SIGMA B9643, Sigma, St. Louis, MO]) and reagent B (copper [II] sulfate pentahydrate 4% [SIGMA C2284, Sigma]) was added. The microplate was incubated for 30 min and absorbance at 562 nm (BIO-RAD ELISA reader model 3550, Hercules, CA) was compared with a protein bovine serum albumin (BSA) standard curve (1 mg/mL).

After the protein concentration of each lysate was adjusted to 60 µg/mL, 1 µL of lysate and 1 µL each from dilutions 1:5 (12 µg/mL), 1:25 (2.4 µg/mL), and $\leq$1:125 (0.48 µg/mL) were spotted onto two nitrocellulose membranes (Millipore, Bedford, MA) for dot-blot analysis. Each membrane was immersed in 1% BSA/PBST blocking buffer (0.05% Tween 20, 1 mM EDTA pH 8.5, 1% BSA in sterile PBS, incubated at room temperature for 1 hr, and washed three times with sterile PBS. Membranes were then immersed in a dilution of anti-PspA rabbit polyclonal antibodies (1:5,000 in blocking buffer) and incubated at room temperature for 1 hr. Rabbit antisera for families 1 and 2 were processed on separate dot blots. The membranes were then washed three times with sterile PBS and incubated with a biotinylated goat anti-rabbit antibody diluted 1:3,000. The final incubation was with streptavidin-conjugated alkaline phosphatase diluted 1:3,000 and incubated for 1 hr, followed by another washing step. Alkaline phosphatase staining was developed with NBT solution (2 mg nitroblue tetrazolium, 10 mg BCIP [5-bromo-4-chloro-3-indolyl phosphate, p-toluidine salt], 200 µL dimethyl sulfoxide in 20 mL Tris HCl, pH 8.8) with constant shaking until control dots appeared purple. Assignment of the serologic dot-blot results was based on the highest titer of each lysate that reacted with the dilution of the anti-family 1 and anti-family 2 antiserum. BG9739 (clade 1) and EF10197 (clade 2) were used as reference strains for serologic family 1 typing. AC122 (clade 3), BG11703 (clade 4) and ATCC6303 (clade 5) were used as reference strains for typing family 2 (16).

### Results

PCR products ranged from 960 to 1,000 bp for family 1, and 1,200 to 1,400 bp for family 2 (Figure). Although all lysates of family 1 strains reacted with the family 1 antiserum at 1:5,000 dilution, the antiserum cross-reacted weakly with family 2 lysates, which could generally be detected at a $\leq$1:25 dilution but not at higher dilutions. The family 2 antiserum reacted only with the PspAs of family 2 strains, regardless of the dilution. Nevertheless, the combination of the two techniques could reliably detect the PspA family of all strains. The PspA PCR and the PspA serologic dot-blot techniques correlated in 100% of cases.

Of the 40 isolates studied, 25 (62.5%) were family 1 PspA (Table). Family 1 PspAs were found among isolates with capsular types 14, 6B, 23F, 5, 19F, 1, 8, and 35. All invasive isolates capsular type 6B, 5, and 19F were family 1. Fourteen (35%) isolates were family 2, including strains of capsular types 14, 23F, 9, 3, 8, and 35. The relative distribution of family 1 isolates to family 2 isolates did not fluctuate substantially over the 4-year period. Isolate Co-29, which was capsular type 1, was the only one (2.5%) of the 40 that was neither family 1 or family 2. The remaining four isolates of capsular type 1 were family 1.

Table. *Streptococcus pneumoniae* PspA families 1 and 2 from 40 Colombian isolates

| Isolates (Code INS[a]) | Date | Source | Capsular type | PCR & dot blot |
|---|---|---|---|---|
| Co-1(2) | 17/12/93 | Blood | 14 | Family 1 |
| Co-2 (125) | 12/12/94 | CSF | 14 | Family 2 |
| Co-3 (146) | 25/01/95 | Blood | 14 | Family 2 |
| Co-4 (309) | 17/01/96 | PLF | 14 | Family 1 |
| Co-5 (E-212) | 16/06/98 | CSF | 14 | Family 2 |
| Co-6 (95) | 17/06/93 | CSF | 6B | Family 1 |
| Co-7 (20) | 19/06/94 | Blood | 6B | Family 1 |
| Co-8 (149) | 04/02/95 | CSF | 6B | Family 1 |
| Co-9 (322) | 10/02/96 | CSF | 6B | Family 1 |
| Co-10 (E-10) | 03/05/96 | Blood | 6B | Family 1 |
| Co-11(14) | 20/04/94 | PLF | 23F | Family 2 |
| Co-12 (23) | 22/06/94 | CSF | 23F | Family 2 |
| Co-13 (152) | 15/02/95 | CSF | 23F | Family 1 |
| Co-14 (E-8) | 03/05/96 | CSF | 23F | Family 1 |
| Co-15 (E-99) | 19/03/97 | CSF | 23F | Family 2 |
| Co-16 (7) | 25/03/94 | PLF | 5 | Family 1 |
| Co-17 (106) | 10/11/94 | Blood | 5 | Family 1 |
| Co-18 (177) | 06/04/95 | Blood | 5 | Family 1 |
| Co-19 (316) | 24/01/96 | Blood | 5 | Family 1 |
| Co-20 E-220) | 24/07/98 | CSF | 5 | Family 1 |
| Co-21(10) | 25/04/94 | CSF | 19F | Family 1 |
| Co-22 (179) | 08/04/95 | CSF | 19F | Family 1 |
| Co-23 (318) | 27/01/96 | Blood | 19F | Family 1 |
| Co-24 (E-124) | 04/07/97 | CSF | 19F | Family 1 |
| Co-25 (E-159) | 23/10/97 | CSF | 19F | Family 1 |
| Co-26 (5) | 19/03/94 | Blood | 1 | Family 1 |
| Co-27 (99) | 04/11/93 | CSF | 1 | Family 1 |
| Co-28 (165) | 01/03/95 | Blood | 1 | Family 1 |
| Co-29 (315) | 26/01/96 | PLF | 1 | 0[b] |
| Co-30 (E-107) | 16/04/97 | Blood | 1 | Family 1 |
| Co-31(51) | 04/09/94 | PLF | 9V | Family 2 |
| Co-32 (E-24) | 14/06/96 | CSF | 9N | Family 2 |
| Co-33 (119) | 25/11/94 | Blood | 3 | Family 2 |
| Co-34 (E-140) | 02/09/97 | CSF | 3 | Family 2 |
| Co-35 (216) | 06/06/95 | Blood | 8 | Family 2 |
| Co-36 (E-135) | 13/08/97 | CSF | 8 | Family 1 |
| Co-37 (E-66) | 07/11/96 | CSF | 4 | Family 2 |
| Co-38 (300) | 18/12/95 | Blood | 35B | Family 1 |
| Co-39 (E-25) | 14/06/96 | CSF | 35B | Family 2 |
| Co-40 (320) | 06/02/96 | CSF | 35F | Family 2 |

[a]INS = Instituto Nacional de Salud; PCR = polymerase chain reaction; CSF = cerebrospinal fluid; PLF = pleural fluid.

[b]Co-29 did not amplify or react with either family.

Figure. Polymerase chain reaction for PspA families 1 and 2: lanes 1 and 2 were controls for families 1 and 2, respectively. Lanes 3 to 7 and 9 (Co-24, Co-25, Co-26, Co-27, Co-28, Co-29, Co-30 isolates) were family 1. Lane 8 (Co-29) did not amplify with either family. The molecular weight was 1-kb ladder DNA (Promega).

### Conclusion

A vaccine composed of PspA is hypothesized to protect against invasive disease and also eliminate the carriage state. Yamamoto et al. (25) reported that intranasal administration of PspA in mice together with a nontoxic adjuvant (mCT S61F) is an effective mucosal vaccine against pneumococcal infection. In another study, active immunization with PspA reduced the signs of purulent otitis media in rats, although the challenge strain contained a PspA that differed from the immunogen (26). More recently, immune sera from human volunteers immunized with PspA protected against fatal pneumococcal infection in mice (27).

All the Colombian isolates were invasive, and all but one belonged to PspA families 1 or 2. This finding is relevant to efforts to develop PspA into a human vaccine component. The distribution of the Colombian isolates between PspA families 1 and 2 did not differ substantially from that observed for isolates from North America and Europe (16). Therefore, a vaccine formulation including these two families might cover isolates from both North and South America with equal effectiveness. Latin America has a varied distribution of capsular serotypes (17-19), which lessens the potential for effectiveness of the heptavalent conjugate vaccine recently approved in the United States (28).

We have obtained information about pulsed-field gel electrophoresis and penicillin-binding protein patterns and capsular types of Colombian strains with diminished susceptibility to penicillin (20,29,30). Characterizing PspA families of these penicillin-resistant strains and examining representatives of different multiresistant international clones will be of interest for future studies.

### Acknowledgments

We thank J. King, A. Swift, S. Chambers, and M. Golden for teaching us the techniques required for this study and H. Roch for careful reading of the manuscript.

This work was partially funded through the Gorgas Memorial Institute and from the U. S. Agency for International Development through the Harvard Institute for International Development. Additional financial support was provided by the Pan American Health Organization and the Canadian International Development Agency.

Ms. Vela is a scientific investigator in the Microbiology Group at Instituto Nacional de Salud in Bogotá, Colombia. Her research interests focus on resistant and multidrug-resistant *Streptococcus pneumoniae* recovered from children <5 years of age.

### References

1. Hoges RG, MacLeod CM. Epidemic pneumococcal pneumoniae. I. Description of the epidemic. Am J Hyg 1946;44:183-92.
2. Klein JO, Teele DW, Sloyer JL, Ploussard JH, Howie V, Makela PH, et al. Use of pneumococcal vaccine for prevention of recurrent episodes of otitis media. In: Robbins JB, Sadoff JC, editors. Bacterial vaccines. New York: Thime-Stratton Press; 1982. p. 305-10.
3. Cowan MJ, Ammann AJ, Wara DW, Howie VM, Schultz L, Doyle N, et al. Pneumococcal polysaccharide immunization in infants and children. Pediatrics 1978;62:721-7.
4. Sniadack DH, Schwartz B, Lipman H. Potential interventions for the prevention of childhood pneumoniae: geographic and temporal differences in serotype and serogroup distribution of sterile site pneumococcal isolates from children-implications for vaccine strategies. Pediatr Infect Dis J 1995;14:503-10.
5. Gray BM, Dillion HC, Briles DE. Epidemiological studies of *Streptococcus pneumoniae* in infants: development of antibody to phosphocholine. J Clin Microbiol 1983;18:1102-7.
6. Briles DE, Creech T, Swiartlo E, Dillard JP, Smith P, Benton KA, et al. Pneumococcal diversity: considerations for new vaccine strategies with emphasis on pneumococcal surface protein A (PspA). Clin Microbiol Rev 1998;11:645-57.
7. McDaniel LS, Yother J, Vijayakumar M, McGarry L, Guild WR, Briles DE. Use of insertional inactivation to facilate studies of biological properties of pneumococcal surface protein A (PspA). J Exp Med 1987;165:381-94.
8. Crain MJ, Waltman II WD, Turner JS, Yother J, Talkington DF, McDaniel LS, et al. Pneumococcal surface protein A (PspA) is serologically highly variable and is expressed by all clinically important capsular serotypes of *Streptococcus pneumoniae*. Infect Immun 1990;58:3293-9.
9. McDaniel LS, Scott G, Kearney JF, Briles DE. Monoclonal antibodies against protease-sensitive pneumococcal antigens can protect mice from fatal infection with *Streptococcus pneumoniae*. J Exp Med 1984;160:386-97.
10. McDaniel LS, Sheffield JS, Delucchi P, Briles DE. PspA, a surface protein of *Streptococcus pneumoniae*, is capable of eliciting protection against pneumococci of more than one capsular serotype. Infect Immun 1991;59:222-8.
11. Briles DE, Ades E, Paton JC, Sampson JS, Carlone JM, Huebner RC, et al. Intranasal immunization of mice with a mixture of the pneumococcal proteins PsaA and PspA is highly protective against nasopharyngeal carriage of *Streptococcus pneumoniae*. Infect Immun 2000;68:796-800.
12. Wu H-Y, Nahm M, Guo Y, Russell M, Briles DE. Intranasal immunization of mice with PspA (pneumococcal surface protein A) can prevent intranasal carriage and infection, and sepsis with *Streptococcus pneumoniae*. J Infect Dis 1997;175:893-6.
13. McDaniel LS, McDaniel DO, Hollingshead SK, Briles DE. Comparison of the PspA sequence from *Streptococcus pneumoniae* EF5668 to the previously identified PspA sequence from strain Rx1 and ability of PspA from EF5668 to elicit protection against pneumococci of different capsular types. Infect Immun 1998;66:4748-54.
14. Yother J, Briles DE. Structural properties and evolutionary relationships of PspA, a surface protein of *Streptococcus pneumoniae*, as revealed by sequence analysis. J Bacteriol 1992;174:601-9.
15. McDaniel LS, Ralph BA, McDaniel DO, Briles DE. Localization of protection-eliciting epitopes on PspA of *Streptococcus pneumoniae* between amino acid residues 192 and 260. Microb Pathog 1994;17:323-37.
16. Hollingshead SK, Becker R, Briles DE. Diversity of PspA: mosaic genes and evidence for past recombination in *Streptococcus pneumoniae*. Infect Immun 2000;68:5889-900.
17. Di Fabio J-L, Homma A, De Quadros C. Pan American Health Organization epidemiological surveillance network for *Streptococcus pneumoniae*. Microb Drug Resist 1997;3:131-3.
18. Castañeda E, Leal AL, Castillo O, de la Hoz F, Vela MC, Arango M, et al. Distribution of capsular types and antimicrobial susceptibility of invasive isolates of *Streptococcus pneumoniae* in Colombian children. Pneumococcal Study Group in Colombia. Microb Drug Resist 1997;3:147-52.
19. Kertesz D, Di Fabio JL, Brandileone MC, Castañeda E, Echániz G, Heitman I, et al. Invasive *Streptococcus pneumoniae* infection in Latin-America children: result of the Pan-American Health Organization surveillance study. Clin Infect Dis 1998;26:1355-61.
20. Vela MC, Fonseca N, Di Fabio JL, Castañeda E. Presence of international multiresistant clones of *Streptococcus pneumoniae* in Colombia. Microb Drug Resist. In press 2001.
21. Facklam RR, Washington JA II. *Streptococcus* and related catalase-negative Gram positive cocci. In: Balows A, Hausler WJ Jr, Hermann KL, Isenberg HD, Shadomy HJ, editors. Manual of clinical microbiology. 5th ed. Washington: American Society for Microbiology; 1991:238-57.
22. Pitcher DG, Saunders NA, Owen RJ. Rapid extraction of bacterial genomic DNA with guanidium thyocyanate. Lett Appl Microbiol 1989;8:151-6.

23. Swiatlo E, Brooks-Walter A, Briles DE, McDaniel LS. Oligonucleotides identify conserved and variable regions of pspA and pspA-like sequences of *Streptococcus pneumoniae*. Gene 1997;188:279-84.
24. Smith PK, Krohn RI, Hermanson GT, Mallia AK, Gartner FH, Provenzano MD, et al. Measurement of protein using bicinchonicic acid. Anal Biochem 1985;150:76-85.
25. Yamamoto M, Briles DE, Yamamoto S, Ohmura M, Kiyono H, McGhee JR. A nontoxic adjuvant for mucosal immunity to pneumococcal surface protein A. J Immunol 1998;161:4115-21.
26. White P, Hermansson A, Svanborg C, Briles DE, Prellner K. Effects of active immunization with a pneumococcal surface protein (PspA) and of locally applied antibodies in experimental otitis media. ORL J Otorhinolaryngol Relat Spec 1999;4:206-11.
27. Briles DE, Hollingshead SK, King JE, Swift A, Braun P, Ferguson LM, et al. Immunization of human volunteers with recombinant PspA elicits antibodies that passively protect mice. ORL J Otorhinolaryngol Relat Spec 1999;4:206-11.
28. Black S, Shinefield H, Fireman B, Lewis E, Ray P, Hansen JR, et al. Efficacy, safety and immunogenicity of heptavalent pneumococcal conjugate vaccine in children. Northern California Kaiser permanent vaccine study center group. Pediatr Infect Dis J 2000;22:187-95.
29. Castañeda E, Peñuela I, Vela MC, the Colombian Pneumococcal Study Group, Tomasz A. Penicillin-resistant *Streptococcus pneumoniae* in Colombia: presence of international epidemic clones. Microb Drug Resist 1998;4:233-9.
30. Tomasz A, Corso A, Severina EP, Echaniz-Aviles G, Brandileone MC, Camou T, et al. Molecular epidemiologic characterization of penicillin-resistant *Streptococcus pneumoniae* invasive pediatric isolates recovered in six Latin-American countries: an overview. PAHO/Rockefeller University Workshop. Microb Drug Resist 1998;4:195-207.
